# Association between cooking patterns and the prevalence of hyperlipidemia in Eastern China

**DOI:** 10.1186/s12889-023-17549-9

**Published:** 2024-01-03

**Authors:** Bin Cui, Wen Kai Yuan, Linda Dong-Ling Wang, Fu Rong Wang, Jing Peng, Jian Ying Ma, Xiang Chen, Mei Yin Xu, Jun Ke, Yi Tian

**Affiliations:** 1 Business School of Yangzhou University, Yangzhou, Jiangsu Province 225001 China; 2 Jiangsu Key Laboratory of Zoonosis, Yangzhou University, Yangzhou, 225001 China; 3 Jiangsu Co-Innovation Center for Prevention and Control of Important Animal Infectious Diseases and Zoonoses, Yangzhou University, Yangzhou, Jiangsu Province 225001 China; 4 Institute of Translational Medicine, Medical College, Yangzhou University, Yangzhou, Jiangsu Province 225001 China; 5 Jiangsu Key Laboratory of Experimental & Translational Non-Coding RNA Research, Yangzhou Uni-versity, Yangzhou, Jiangsu Province 225001 China; 6 School of Public Health of Yangzhou University, Yangzhou, Jiangsu Province 225001 China; 7 School of Tourism, Cuisine of Yangzhou University, Yangzhou, Jiangsu Province 225001 China

**Keywords:** Cooking patterns, Hyperlipidemia, Eastern China

## Abstract

**Background:**

Hyperlipidemia is a major risk factor for many diseases. Previous studies have shown that diet is closely associated with hyperlipidemia. However, the relationship between cooking methods and hyperlipidemia remains unclear. The objective of this study was to identify the major cooking patterns existing in the Eastern Chinese population and evaluate their association with the prevalence of hyperlipidemia.

**Methods:**

We interviewed 4,710 residents in Eastern China regarding the consumption frequency of each cooking method when they prepare food at home or when eating out and regarding the prevalence of hyperlipidemia. Factor analysis, Chi-square tests, analysis of variance, and binary logistic regression analysis were used to identify the cooking patterns and analyze the characteristics of participants’ categories of cooking patterns and the relationship between different cooking patterns and prevalence of hyperlipidemia.

**Results:**

Three major cooking patterns were identified: Traditional Chinese, Bland (little or no oil is used to process the food), and High-temperature cooking patterns. After controlling for potential confounders, participants in the highest quartile of the Bland cooking pattern had lower odds of hyperlipidemia than those in the lowest quartile. Nevertheless, no significant associations were observed between the Traditional Chinese and High-temperature cooking patterns and the prevalence of hyperlipidemia.

**Conclusions:**

This study confirms the association between cooking patterns and the prevalence of hyperlipidemia and indicates that the Bland cooking pattern is associated with a reduced prevalence of hyperlipidemia.

**Supplementary Information:**

The online version contains supplementary material available at 10.1186/s12889-023-17549-9.

## Background

Hyperlipidemia is a major risk factor for cardiovascular disease (CVD), which is the leading cause of morbidity and mortality globally [1]. In addition, recent studies have shown that hyperlipidemia is also a risk factor for bladder cancer [2], breast cancer [3], and enlargement of the prostate [4].

According to the Annual Report on Cardiovascular Health and Diseases in China (2019), total cholesterol (TC), triglycerides (TG), and low-density lipoprotein cholesterol (LDL-C) significantly increased in Chinese adults over the age of 18 between 2002 and 2015, while high-density lipoprotein cholesterol (HDL-C) significantly decreased. Moreover, the overall prevalence of dyslipidemia (defined as the presence of any type of dyslipidemia) in Chinese individuals ≥ 18 years of age was 8.6%, 34.0%, 39.91%, and 40.4% in 2002, 2010, 2011, and 2012, respectively, showing an increasing trend [5].

Diet is closely related to hyperlipidemia [6, 7], and dietary patterns have been significantly associated with hyperlipidemia [8, 9]. Cooking methods modify the organoleptic conditions of foods, making them more palatable and influencing the bioavailability of nutrients, vitamins, and minerals [10]. Some cooking methods such as frying not only increase fat and energy content but also modify food composition. For example, frying food causes water to be replaced by fat, and deep frying results in an increase in trans-fatty acids [11]. Studies have found that frequent consumption of fried foods is associated with a higher risk of hypertension [12], and the social business cooking patterns have shown a relationship with inflammatory and cardio-metabolic health biomarkers [13]. However, the relationship between cooking patterns and hyperlipidemia is unclear.

Over the years, the Chinese people have developed many distinct and unique cooking methods. Data show that there are more than 30 kinds of basic cooking techniques commonly used in China [14]. However, as far as we have been able to determine, no previous study has assessed the association between cooking patterns and the prevalence of hyperlipidemia among the Chinese population.

Eastern China encompasses Jiangsu, Shandong, Anhui, Zhejiang, and Fujian Provinces, as well as the city of Shanghai, and its regional cuisine holds an important position in the culture of Chinese cuisine. Eastern China is home to five of China’s eight major cuisines [15]. Therefore, studies related to cooking performed in Eastern China are useful for guiding decision-making and policymaking nationwide.

The present study identified the major cooking patterns existing in the Eastern Chinese population and evaluated their association with the prevalence of hyperlipidemia.

## Materials and methods

### Study population

**Fig. 1:**
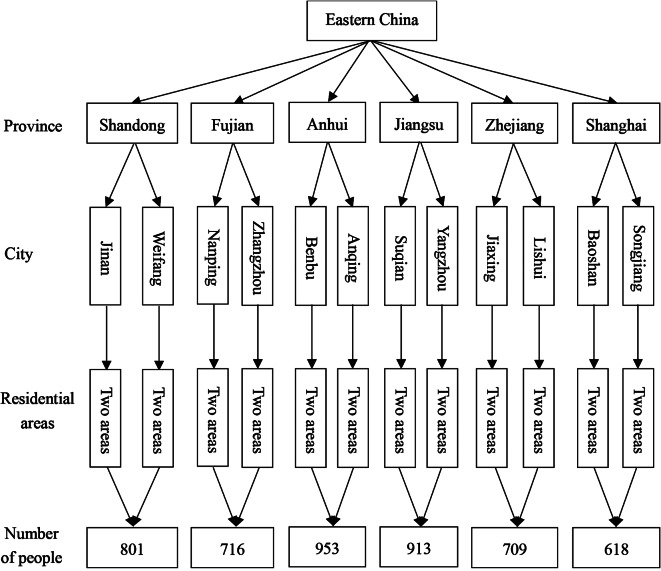
Flow chart showing the sampling process

The present cross-sectional study was conducted in Eastern China from March to June 2021. The sample was selected by a stratified cluster random-sampling method. Two cities were randomly selected from each province. In each selected city, two residential areas were randomly selected for sampling. Between180 and240 residents were recruited from each residential area (Fig. 1). Those who agreed to participate completed a face-to-face interview using a standardized questionnaire (see Appendix) conducted by five trained research assistants. Interviews were conducted at the entrance of each residential area in the afternoon at the end of a normal workday. The average interview lasted approximately 15 min per participant. Upon completion of the interview, each participant received a small gift worth approximately 5 Chinese yuan. A total of 4,710 participants completed interviews, for a response rate of 98.1%. Only 90 residents refused to participate or did not complete the interview, citing lack of time or no recent routine physical examination [16].

### Assessment of cooking methods

Based on *The Comprehensive Cooking Techniques of China* [14] and traditional cooking techniques frequently used by residents of the surveyed provinces and cities in East China [15], we summarized 15 major cooking methods. These consisted of stir-fry and sauté (put a small amount of oil in a pan and cook food quickly over high heat, stirring and turning the pan), boiling (to cook in water or liquids when the temperature is at boiling point), steaming (to cook using vapor), pan-frying (to cook food in a pan with the minimum amount of oil), roasting (to cook in an oven using hot air or radiation, which cooks the food evenly), deep-frying (to cook food in hot oils when the food is totally immersed), stewing (to boil slowly or simmering in a liquid for a long period of time at low heat), marinated in spirits (to soak food in liquor or rice wine for sterilization through pickling), blanching (food is put in boiling water, turned and removed in time, and then sautéed), poaching (to cook the food in boiling water for a short time, remove and pour into a bowl, add fresh clear soup, then season), mixed in soy sauce (to process food into strips or sheets, put into boiling water or hot oil, remove and season), stir-frying and fast-sautéing (to cook small foods in boiling oil or water quickly (about 10 s)), simmer and keep the shape (arrange the raw materials in a neat pattern, add the right amount of stock and condiments and gently heat until mature, keeping the shape), deep-fry first, then season with sauce (fry food materials over high heat until yellow and stiff, then season with sauce), marinated in rice wine (marinate raw materials in rice wine).

Participants were asked to report their adoption and consumption frequency of each cooking method when they prepare food at home or eating out in the previous 12 months (Never eating = 1, Eating occasionally = 2, Sometimes eating = 3, Often eating = 4, and Eating every day = 5).

### Identification of cooking patterns

The Kaiser-Meyer-Olkin (KMO) measure of sampling adequacy and Bartlett’s test of sphericity were used to evaluate the adequacy of correlation matrices with the data. Factor analysis (principal component) was used to derive the major cooking patterns. The factors were rotated using orthogonal transformation (varimax rotation) to achieve uncorrelated factors and greater interpretability. The number of factors retained was determined by an eigenvalue ≥ 1. Cooking groups with an absolute factor loading ≥ 0.6 were considered important contributors to this pattern.

The labeling of cooking patterns was based on the interpretation of cooking methods with high factor loading on each pattern. Quartiles based on factor scores were determined for each cooking pattern (Q1 represented a low intake of the food by the cooking pattern; Q4 represented a high intake of the food by the cooking pattern).

### Definition of variables

Standing height and body weight were measured without shoes and in light clothes by trained researchers. Body mass index (BMI, kg/m^2^) was calculated as weight in kilograms divided by height in meters squared. Physical activity and hyperlipidemia were obtained through self-reporting. Participants’ physical activity levels were assessed using the International Physical Activity Questionnaire (IPAQ) [17]. The metabolic equivalent of task (MET) level for each reported activity was calculated based on its duration (hours) per week. Participants were then grouped into one of three categories: Light (< 3 MET), Moderate (3–6 MET), or Vigorous (> 6 MET) [18]. Additionally, their hyperlipidemia diagnosis (yes/no) was recorded based on their most recent doctor visit or a routine physical examination within the past year. Participants reported their gender, age, education level, and total monthly household income.

### Statistical analyses

Data were calculated across quartiles for each cooking pattern score and are presented as the mean and standard deviation for continuous variables, or as number and percentage for categorical variables. The Chi-square test was used to assess the differences for categorical variables, while the analysis of variance (ANOVA) was used to describe mean differences for continuous variables. After adjusting for confounders, binary logistic regression analysis was used to estimate the odds ratio (OR) and 95% CI for hyperlipidemia according to quartiles of each cooking pattern score (the first quartile of each pattern was used as the reference group). All statistical analyses were performed using the statistical package SPSS version 21.0, and 2-tailed P-values < 0.05 were considered statistically significant.

## Results

The overall prevalence of hyperlipidemia in this population was 3.8%. In males it was 2.4%, and in females it was 1.4%. The participant characteristics with and without hyperlipidemia are shown in Table 1. There were significant differences between participants with and without hyperlipidemia by gender, age, education, income, physical activity, and BMI.

**Table 1:** Characteristics of participants with and without hyperlipidemia

Variables	Participants with hyperlipidemia (n = 181)	Participants without hyperlipidemia (n = 4529)	Significance
Gender			χ^2^ = 25.86
Male	112 (61.9%)	1937 (42.8%)	P = 0.000
Female	69 (38.1%)	2592 (57.2%)	
Age			χ^2^ = 227.46
≤ 20 years	5 (2.8%)	880 (19.4)	P = 0.000
21–35	6 (3.3%)	1287 (28.4%)	
36–45	47 (26.0%)	1280 (28.3%)	
46–55	79 (43.6%)	829 (18.3%)	
56–65	40 (22.1%)	214 (4.7%)	
66–75	4 (2.2%)	30 (0.7%)	
≥ 76 years	0 (0.0%)	8 (0.2%)	
Education			χ^2^ = 17.95
Primary or below	5 (2.8%)	87 (1.9%)	P = 0.003
Junior high school	15 (8.3%)	640 (14.1%)	
Senior high school	19 (10.5%)	886 (19.6%)	
Three-year college	36 (19.9%)	794 (17.5%)	
Undergraduate college	85 (47.0%)	1704 (37.6%)	
Postgraduate and above	21 (11.6%)	418 (9.2%)	
Monthly family income (Chinese Yuan)			χ^2^ = 27.07
≤ 5000	25 (13.8%)	1276 (28.2%)	P = 0.000
5000–9999	60 (33.1%)	1488 (32.9%)	
10,000–19,999	59 (32.6%)	1040(23.0%)	
20,000–39,999	25 (13.8%)	439 (9.7%)	
40,000–80,000	3 (1.7%)	155 (3.4%)	
≥ 80,001	9 (5.0%)	131 (2.9%)	
Physical activity			χ^2^ = 21.31
Light	107(59.1%)	1896(41.9%)	P = 0.000
Moderate	58(32.0%)	2012 (44.4%)	
Vigorous	16(8.8%)	621(13.7%)	
BMI (mean value)	24.17	22.67	T = − 5.36 (P = 0.000)

Both the Kaiser-Meyer-Olkin index (0.901) and Bartlett’s test (P < 0.001) indicated that the correlation among the variables was sufficiently strong for a factor analysis. Factor analysis identified three cooking patterns: the Traditional Chinese cooking pattern (high-frequency consumption of food Marinated in spirits, Blanching, Poaching, Mixed in soy sauce, Stir-frying and fast-sautéing, Simmer and keep the shape, Deep-fry first, then season with sauce, and Marinated in rice wine; the Bland cooking pattern (high-frequency consumption of Stir-frying and sautéing, Boiling, Steaming, and Stewing); and the High-temperature cooking pattern (high-frequency consumption of Pan-frying, Roasting, and Deep-frying). These patterns explained 30.0%, 15.2%, and 14.5% of the cooking method consumption variance. The factor loading matrices for the cooking patterns are provided in Table 2.

**Table 2:** Factor-loading matrix for the three cooking patterns

	Cooking patterns		
	Traditional Chinese	Bland cooking	High-temperature cooking
Stir-frying and sauté	-	0.691	-
Boiling	-	0.838	-
Steaming	-	0.709	-
Stewing	-	0.602	-
Pan-frying	-	-	0.732
Roasting	-	-	0.778
Deep-frying	-	-	0.815
Marinated in spirits	0.684	-	-
Blanching	0.687	-	-
Poaching	0.785	-	-
Mixed in soy sauce	0.785	-	-
Stir-frying and fast-sauté	0.705	-	-
Simmer and keep the shape	0.769	-	-
Deep-fry first, then season with sauce	0.580	-	-
Marinated in rice wine	0.733	-	-
Variance explained (%)	30.0	15.2	14.5
Cumulative variance explained (%)	30.0	45.2	59.7
Initial Eigenvalue	5.52	2.28	1.15

The characteristics of the study participants by quartiles of cooking pattern scores are shown in Table 3. Participants within the top quartile of the Traditional Chinese pattern were male and younger, with higher education and monthly income, and undertook moderate physical activity; more people had no hyperlipidemia and had higher BMI compared with participants in the lowest quartile. Compared with those in the lowest quartile, participants in the highest quartile of the Bland cooking pattern were female and older, had higher education and monthly income, and undertook light physical activity. Furthermore, we also found that participants in the highest quartile of the High-temperature cooking pattern were male and younger, had lower education and monthly income, undertook moderate physical activity, had no hyperlipidemia, and had lower BMI than those in the lowest quartile.

**Table 3:** Characteristics of the study participants by quartile (Q) categories of cooking pattern scores

	Traditional Chinese					Bland cooking					High-temperature cooking				
	Q1 (lowest) (n1177)		Q4 (highest) (n1177)			Q1 (lowest) (n1177)		Q4 (highest) (n1179)			Q1 (lowest) (n1176)		Q4 (highest) (n1216)		
	Mean or n	SD or%	Mean or n	SD or%	P*	Mean or n	SD or%	Mean or n	SD or%	P*	Mean or n	SD or%	Mean or n	SD or%	P*
Gender					< 0.001					< 0.001					< 0.05
Male	433	36.8	587	49.9		558	47.4	435	36.9		485	41.2	571	47.0	
Female	744	63.2	590	50.1		619	52.6	744	63.1		691	58.8	645	53.0	
Age					< 0.001					< 0.001					< 0.001
≤ 20 years	197	16.8	338	28.7		267	22.7	150	12.7		95	8.1	388	31.9	
21–35	324	27.6	335	28.5		392	33.3	296	25.1		232	19.7	403	33.2	
36–45	357	30.4	292	24.8		300	25.5	322	27.3		354	30.1	252	20.7	
46–55	220	18.7	155	13.2		188	16.0	293	24.9		342	29.1	139	11.4	
56–65	66	5.6	50	4.2		27	2.3	95	8.1		130	11.1	26	2.1	
66–75	11	0.9	6	0.5		3	0.3	20	1.7		21	1.8	5	0.4	
≥ 76 years	1	0.1	1	0.1		0	0.0	2	0.2		2	0.2	2	0.2	
Education					< 0.001					0.001					< 0.05
Primary or below	27	2.3	20	1.7		32	2.7	16	1.4		24	2.0	24	2.0	
Junior high school	225	19.1	146	12.4		179	15.2	142	12.0		153	13.0	137	11.3	
Senior high school	191	16.2	231	19.6		249	21.2	201	17.0		213	18.1	251	20.6	
Three-year college	192	16.3	216	18.4		206	17.5	211	17.9		200	17.0	225	18.5	
Undergraduate college	424	36.0	457	38.8		411	34.9	473	40.1		479	40.7	463	38.1	
Postgraduate and above	118	10.0	107	9.1		100	8.5	136	11.5		107	9.1	116	9.5	
Monthly family income (Chinese Yuan)					< 0.001					< 0.001					< 0.001
≤ 5000	406	34.5	334	28.4		379	32.2	283	24.0		267	22.7	366	30.1	
5000–9999	378	32.1	366	31.1		401	34.1	362	30.7		378	32.1	394	32.4	
10,000–19,999	252	21.4	249	21.2		240	20.4	313	26.5		346	29.4	251	20.6	
20,000–39,999	91	7.7	135	11.5		97	8.2	143	12.1		117	9.9	104	8.6	
40,000–80,000	27	2.3	40	3.4		26	2.2	51	4.3		42	3.6	48	3.9	
≥ 80,001	23	2.0	53	4.5		34	2.9	27	2.3		26	2.2	53	4.4	
Hyperlipidemia					< 0.001					0.22					< 0.05
no	1137	96.6	1152	97.9		1138	96.7	1135	96.3		1128	95.8	1190	97.9	
yes	40	3.4	25	2.1		39	3.3	44	3.7		50	4.2	26	2.1	
BMI (kg/m^2^)	22.69	3.74	22.73	3.73	< 0.05	22.70	3.93	22.79	3.70	0.55	23.00	3.44	22.22	3.94	< 0.001
Physical activity					< 0.001					< 0.001					< 0.001
Light	490	41.6	466	39.6		429	36.4	565	47.6		570	48.4	488	40.1	
Moderate	484	41.1	564	47.9		567	48.2	485	41.1		460	39.0	579	47.6	
Vigorous	203	17.2	147	12.5		181	15.4	129	10.9		148	12.6	149	12.3	

The associations between cooking patterns and the prevalence of hyperlipidemia by logistic regression analysis are presented in Table 4. After adjusting for potential confounding variables, participants in the highest quartile of the Bland cooking pattern had lower odds of hyperlipidemia (OR = 0.59, 95% CI 0.37, 0.94; P < 0·05) than those in the lowest quartile. In addition, no significant associations between Traditional Chinese and High-temperature cooking patterns and the prevalence of hyperlipidemia were observed.

**Table 4:** Multivariable-adjusted ORs and P-values for hyperlipidemia across quartile (Q) categories of cooking pattern scores

	Traditional Chinese pattern									
	Q1	Q2			Q3			Q4		
	(reference)	OR	95% CI	P	OR	95% CI	P	OR	95% CI	P
Model 1 (unadjusted)	1.00	1.63	1.089, 2.441	< 0.05	1.31	0.862, 1.999	0.21	0.62	0.371, 1.023	0.06
Model 2 (adjusted)	1.00	1.35	0.890, 2.062	0.16	1.11	0.717, 1.718	0.64	0.61	0.360, 1.034	0.07
Model 3 (adjusted)	1.00	1.29	0.847, 1.972	0.23	1.07	0.690, 1.659	0.76	0.62	0.368, 1.056	0.08
	Bland cooking pattern									
	Q1	Q2			Q3			Q4		
	(reference)	OR	95% CI	P	OR	95% CI	P	OR	95% CI	P
Model 1 (unadjusted)	1.00	1.48	0.978, 2.246	0.06	1.06	0.675, 1.648	0.81	1.13	0.730, 1.756	0.58
Model 2 (adjusted)	1.00	1.21	0.786, 1.872	0.38	0.64	0.401, 1.030	0.07	0.60	0.372, 0.953	< 0.05
Model 3 (adjusted)	1.00	1.24	0.801, 1.915	0.34	0.64	0.398, 1.025	0.06	0.59	0.366, 0.942	< 0.05
	High-temperature cooking pattern									
	Q1	Q2			Q3			Q4		
	(reference)	OR	95% CI	P	OR	95% CI	P	OR	95% CI	P
Model 1 (unadjusted)	1.00	0.92	0.610, 1.381	0.68	1.23	0.838, 1.813	0.29	0.49	0.305, 0.798	< 0.05
Model 2 (adjusted)	1.00	1.53	0.993, 2.356	0.06	2.20	1.457, 3.333	< 0.05	1.10	0.659, 1.833	0.72
Model 3 (adjusted)	1.00	1.51	0.978, 2.325	0.06	2.25	1.484, 3.406	< 0.05	1.16	0.695.1.935	0.57

Model 1: unadjusted; Model 2: further adjusted for gender, age, educational level, and economic income; Model 3: additionally, adjusted for physical activity level and BMI; Q4: the highest quartile of dietary patterns; Q1: the lowest quartile of dietary patterns (reference); and CI: confidence interval

## Discussion

In this study of the Eastern Chinese population, the prevalence of hyperlipidemia was low (3.8%), which may be related to the self-report method used in this survey. We identified three major cooking patterns: Traditional Chinese, Bland, and High temperature. The Bland cooking pattern was inversely associated with the prevalence of hyperlipidemia. To the best of our knowledge, the present study is the first to examine the relationship between major cooking patterns and the prevalence of hyperlipidemia in the Chinese population.

This study found that the proportion of male respondents suffering from hyperlipidemia was significantly higher than that of female respondents, and the proportion of respondents in the 46–55 age group suffering from hyperlipidemia was significantly higher compared to other age groups. This was consistent with Annual Report on Cardiovascular Health and Diseases in China (2019) [5]. Our study further found that the proportion of undergraduate college respondents suffering from hyperlipidemia was significantly higher than that of respondents who attained other education levels, and the proportion of respondents with monthly family income (Chinese Yuan) of 5,000–19,999 suffering from hyperlipidemia was significantly higher than that of respondents at other income levels.

In the present study, the traditional Chinese cooking pattern was characterized by a high-frequency consumption of Marinated in spirits, Blanching, Poaching, Mixed in soy sauce, Stir-frying and fast-sautéed, Simmer and keep the shape, Deep-fry first then season with sauce, and Marinated in rice wine. In our data, no significant association was observed between this pattern and the prevalence of hyperlipidemia, and participants with more Traditional Chinese cooking patterns had a lower rate of hyperlipidemia. One possible explanation is that less oil is used as a medium for heating food in the above cooking methods, although previous studies have shown that lipid intake can be a risk factor for hyperlipidemia [6]. Oil is used to heat food for a shorter time in the cooking method of Mixed in soy sauce and Stir-frying and fast-sautéing cooking methods. Additionally, spirits and rice wine are used to treat foods in the method of Marinated in spirits and Marinated in rice wine methods. There have been no reports of hyperlipidemia associated with small amounts of alcohol used during cooking.

The Bland cooking pattern was characterized by a high-frequency consumption of Stir-frying and sautéing, Boiling, Steaming, and Stewing. Steaming, Boiling, and Stewing mainly rely on water vapors or water to transfer heat [19]. In our analyses, we observed an inverse association between the Bland cooking pattern and the prevalence of hyperlipidemia. Our findings align with those of a previous study that reported a significant association of a high consumption of deep-fried foods and a low intake of steamed, boiled, and raw food with hyperlipidemia [8]. Another related study also showed that boiling and sautéing, brining, and light frying tend to have cardio-metabolic benefits [13]. One possible mechanism for this is that steaming increases the concentration of polyphenols and antioxidants [20], and dietary polyphenols can lower LDL-C levels [21]. Additionally, in a comparison of the effects of steaming, oven cooking, and deep fat-frying on the physicochemical and sensory quality of turkey meat patties, steamed patties showed the lowest shrinkage and fat content [22].

The High-temperature cooking pattern is characterized by the frequent use of pan-frying, roasting, and deep-frying techniques. Frying relies on oil to transfer heat, and roasting typically involves cooking on an open flame or baking using coal, firewood, or charcoal during fire roasting [19]. In our study, no significant association was found between the High-temperature cooking pattern and the prevalence of hyperlipidemia. Our findings contrast with previous studies reporting that a low-cholesterol and low-fat (particularly saturated fat) diet is beneficial for managing hyperlipidemia [23]. In addition, the frequent consumption of fried foods has been linked to a higher prevalence of hypertension [12]. However, other studies have shown that dietary cholesterol consumption is not necessarily associated with dyslipidemia or serum lipids [24] and that a high intake of deep-fried foods has no association with components of dyslipidemia [9]. Moreover, some research reports a lack of association between dietary cholesterol intake with dyslipidemia, hypertriglyceridemia, and HDL-hypocholesterolemia [25]. While the cooking method is different from the diet itself, it can still affect the bioavailability of nutrients, vitamins, and minerals in food [10]. Additionally, the absence of data on the categories of food consumed daily by participants leads us to miss important confounders in the relationships between cooking patterns and hyperlipidemia. Therefore, further study should explore this more deeply.

The present study had some limitations. First, because of the cross-sectional design of the study, we could not assess the causal association between cooking patterns and the prevalence of hyperlipidemia. Therefore, further prospective studies are needed to confirm this finding. Second, hyperlipidemia was established by self-report rather than by a clinician-administered structured diagnostic test, and hyperlipidemia may be inaccurately reported by participants. More studies (including clinical trials) are necessary to evaluate the association between cooking patterns and their association with the incidence of hyperlipidemia. Third, the recall method used in the evaluation of cooking methods may lead to some degree of misclassification. Fourth, although various confounders were considered, we could not discount residual confounding. Finally, the study participants were recruited in Eastern China. Therefore, our results may not be generalizable to the entire Chinese population.

## Conclusions

We identified three major cooking patterns, namely the Traditional Chinese, Bland, and High-temperature cooking patterns. Our results demonstrate that the Bland cooking pattern is associated with a reduced prevalence of hyperlipidemia. Present findings provide further insight into understanding the associations between cooking patterns and hyperlipidemia. Further longitudinal studies and trials are required to elucidate whether a true causal association exists.

### Electronic supplementary material

Below is the link to the electronic supplementary material.

**Supplementary Material 1:** Questionnaire

## Data Availability

The datasets used and/or analyzed during the current study are available from the corresponding author upon reasonable request.
