# Supplementary material for: Association between cooking patterns and the prevalence of hyperlipidemia in Eastern China
Source: BMC Public Health. 2024 Jan 3;24:75. doi: 10.1186/s12889-023-17549-9 (PMC10763481; doi:10.1186/s12889-023-17549-9)
Supplement: Supplementary file 1 — Supplementary Material 1: Questionnaire [file 12889_2023_17549_MOESM1_ESM.docx]

**Supplementary Material (Appendix)**

Questionnaire

**Gender**

□ Male

□ Female

**Age**

□ ≤20 years

□ 21–35

□ 36–45

□ 46–55

□ 56–65

□ 66–75

□ ≥76 years

**Height**

**Weight**

**Education**

□ Primary or below

□ Junior high school

□ Senior high school

□ Three-year college

□ Undergraduate college

□ Postgraduate and above

**Monthly family income (Chinese Yuan)**

□ ≤5000

□ 5000–9999

□ 10000–19999

□ 20000–39999

□ 40000–80000

□ ≥80001

**The physical activity level**

□ Light

□ Moderate

□ Vigorous

**Whether you were diagnosed with hyperlipidemia at your most recent doctor visit or recent routine physical examination**

□ Yes

□ No

**How often do you adopt or consume each cooking method when you prepare food at home or eating out as follows?**

| Cooking method | Never eating | Eating once in a while | Sometimes eating | Often eating | Eating every day |
| --- | --- | --- | --- | --- | --- |
| Stir-frying and sauté | □ | □ | □ | □ | □ |
| Boiling | □ | □ | □ | □ | □ |
| Steaming | □ | □ | □ | □ | □ |
| Stewing | □ | □ | □ | □ | □ |
| Pan-frying | □ | □ | □ | □ | □ |
| Roasting | □ | □ | □ | □ | □ |
| Deep-frying | □ | □ | □ | □ | □ |
| Marinated in spirits | □ | □ | □ | □ | □ |
| Blanching | □ | □ | □ | □ | □ |
| Poaching | □ | □ | □ | □ | □ |
| Mixed in soy sauce | □ | □ | □ | □ | □ |
| Stir-frying and fast-sauté | □ | □ | □ | □ | □ |
| Simmer and keep the shape | □ | □ | □ | □ | □ |
| Deep-fry first, then season with sauce | □ | □ | □ | □ | □ |
| Marinated in rice wine | □ | □ | □ | □ | □ |
